# Premenstrual Syndrome, Anxiety, and Depression Among Menstruating Rural Adolescent Girls: A Community-Based Cross-Sectional Study

**DOI:** 10.7759/cureus.50385

**Published:** 2023-12-12

**Authors:** Priya Mann, Pradeep TS

**Affiliations:** 1 Community Medicine, Sri Devaraj Urs Medical College, Sri Devaraj Urs Academy of Higher Education and Research, Kolar, IND

**Keywords:** depression, anxiety, premenstrual dysphoric disorder, premenstrual tension, adolescent girls

## Abstract

Introduction

Menstrual periods in young females can add a new challenge to the already difficult adolescent transition period. Menstrual health concerns can have extreme physical and psychosocial impacts on adolescent girls. Premenstrual syndrome (PMS) and premenstrual dysphoric disorder (PMDD) are extremely common yet underestimated. Depression in adolescents is a mental and emotional disorder. The objective of the study was to find out the prevalence of PMS, PMDD, anxiety, and depression among rural menstruating adolescent girls and the factors associated with it.

Methods

This was a community-based cross-sectional study carried out among 20 rural schools for the period of one year. Sample size was calculated based on previous research. Adolescent females who had menstruated for one year were included and those with primary amenorrhea and previously diagnosed mental health abnormalities were excluded. Becks' Depression Inventory, Hamilton Anxiety Scale, and Premenstrual Symptoms Screening Tool (PSST-A) were used. Data were collected by interview technique and entered in an Excel sheet (Microsoft Corporation, Redmond, Washington, United States), and analyzed using IBM SPSS Statistics for Windows, Version 22.0 (Released 2013; IBM Corp., Armonk, New York, United States).

Results

Out of 430 rural adolescent girls, 180 (41.9%) were 15 years, 277 (64.4%) belonged to nuclear families, 236 (54.9%) exercised less than 30 minutes, 144 (33.5%) had menarche at the age of 13 years, 288 (67%) had regular cycles, 266 (61.9%) had moderate flow during the menstrual cycle, 302 (70.2%) had a flow duration of less than seven days, and 243 (56.5%) had dysmenorrhea. Thirty-eight (8.8%) girls had PMDD and 75 (17.4%) had PMS. Age, family status, severity of menstrual flow, duration of cycle, and presence of dysmenorrhea, depression, and anxiety had a statistically significant association with PMDD. The class/grade in which studying, cycle regularity, flow during the menstrual cycle, duration of the cycle, dysmenorrhea, anxiety, and depression status had a statistically significant association with PMS.

Conclusions

The menstrual cycle's impact on the mental health of rural adolescent girls should not be ignored and schools can be instrumental in improving their quality of life. Regular counselling and mental health supervision by school teachers and peer groups can be beneficial.

## Introduction

According to the World Health Organisation (WHO), the age group of 10-19 years is said to be adolescence. This age group signifies the transition period from childhood to adulthood, whereby a person experiences tremendous growth and development, which could be physical, psychological, or biological. Compared to boys, girls confront this challenge more, which requires special attention [1]. Menstruation is part of the female reproductive cycle, starting at puberty. The menstrual period can add a new challenge to the already difficult teenage years in girls. A female child is developing physically while simultaneously undergoing rapid growth in psychosocial maturity and other behavioural changes. Many common problems or aggravations can arise with menstrual periods, which typically occur monthly and last six or seven days. Some of these issues can be typical, but others may need to be evaluated by a healthcare professional. Menstruation is a natural process, but it is still taboo in Indian society because it is considered unclean. Menstruation is surrounded by various psychological and religious barriers due to a lack of knowledge about the scientific process of menstruation [2]. Among adolescent girls, fear and shame of menstruation are frequent themes that emerge both in country-specific studies and global reviews [3]. Premenstrual syndrome (PMS) is a cyclic phenomenon of somatic and affective symptoms appearing in the days preceding menstruation and interfering with one’s work or lifestyle, followed by a symptom-free interval [4].

PMS is an extremely common yet underestimated disorder in adolescent school girls, which can adversely affect their emotional well-being [5]. PMS is associated with severe depressive symptoms, irritability, and tension during the premenstrual phase. PMS is associated with both affective and somatic symptoms. Out of many somatic symptoms, depression, anxiety, angry outbursts, irritability, confusion, and social withdrawal are evident [6]. In India, there is still very little focus on adolescent reproductive and sexual health (ARSH) and mental health, which has been linked in the present study. Adolescent depression is a mental and emotional disorder associated with irritability, restlessness, aggressiveness, desire or attempts to run away, feelings of not being accepted, and carelessness with personal hygiene and self-care [7]. Anxiety is a normal human emotion that encompasses behavioural, affective, and cognitive responses to the perception of danger. Recurrent episodes and psychological impairment are apparent with repetitive episodes of anxiety, which can be detrimental. An overly anxious teenage girl might withdraw from activities because she is too scared or anxious, and the same applies to depression. Mid-adolescence girls are more than twice as likely to be diagnosed with a mental health disorder as boys, and menstruation and its disorders definitely have a causal association. Relatively little is known about the link between depressive symptoms, anxiety, and menstrual symptoms, especially in pubertal-aged adolescent girls. Studies have shown that the symptoms of PMS could be outright depression and anxiety. The exacerbation of common medical and mental health disorders at specific phases of the menstrual cycle is a prevalent phenomenon. Although the precise cause of this condition is unclear, complex interactions between the immune and neuroendocrine systems could be one possible reason [8].

The menstrual cycle is also a trigger for the onset of depressive disorders, including premenstrual dysphoric disorder (PMDD), a disorder specific to the luteal phase of the menstrual cycle, and depression associated with the transition to menopause. PMDD is a depressive disorder involving cyclic psychological, cognitive, and somatic symptoms, causing functional impairment within the late luteal phase of the menstrual cycle. Gonadal hormone fluctuations, such as the decline of oestrogen or progesterone, occur in the late luteal phase. Brief recurrent psychotic episodes during puberty and adolescence have been the subject of clinical interest for decades. Mental health issues have been proposed to be related to the menstrual cycle due to the many overlapping psychopathological features [9]. A study undertaken in China by Liu et al. among Chinese female adolescents showed that the onset of menstruation and menstrual problems was related to non-suicidal self-injury, highlighting the importance of menstrual hygiene education and treatment of menstrual problems at schools [10]. In their cohort study, Nillni et al. indicated that higher levels of depressive symptoms were associated with irregular menstrual cycles, suggesting that mental health may be an important determinant of cycle regularity [11]. The authors also showed that the link between adverse mental health exposures and the prevalence of menstrual abnormalities had a statistical significance. However, despite this complexity, recent evidence reveals that biological factors, such as the variation in ovarian hormone levels and particularly decreases in oestrogen, may contribute to the increased prevalence of depression and anxiety in women and that strategies to mitigate decreases in oestrogen levels may be protective.

Evidence of menstrual cycle-dependent fluctuations in psychiatric symptoms is strong, as studies have indicated an increase in psychosis, mania, depression, suicide/suicide attempts, and alcohol use during various phases of menstruation among women, with anxiety and stress appearing to be elevated more generally throughout the luteal phase [12]. The prevalence of PMS among adolescents varies from 10% to 53%, depending on the population studied and diagnostic measures used [13]. Schmidt et al. showed that in women with normal pituitary-gonadal function and PMS, symptoms of PMS occurred in response to normal hormonal changes during the menstrual cycle; thus, in susceptible women, normal levels of gonadal hormones triggered an abnormal response [14]. Against this background, the current study was conducted to determine the prevalence of PMS, PMDD, anxiety, and depression among rural menstruating adolescent girls and to establish the association and correlation between PMT, PMDD, and anxiety and depression among rural menstruating adolescent girls.

## Materials and methods

This was a community-based cross-sectional study conducted from June 1, 2022, to June 1, 2023. Study participants were school-attending adolescent rural girls who had attained menarche. The study was carried out in rural higher primary schools and high schools in Kolar, Karnataka, India. Thirty-six rural schools are found in Kolar taluk (subdivision), out of which 20 were randomly selected. Ethical clearance was obtained from the Ethics Committee, Sri Devaraj Urs Medical College (approval number: DMC/KLR/IEC/754/2022-23). Informed written consent was taken from the parents. Assent from the study participants was obtained by informing them about the benefits and risks involved in the study. Autonomy was maintained by making the participation voluntary and confidentiality was maintained by not recording the participants’ names and identifying details.

Sample size and inclusion criteria

The sample size was calculated based on a previous study by Sarkar et al. [15]. Using prevalence (p) as 61% from their study conducted in West Bengal, error (d) as 5%, with a 95% confidence interval, the sample size was calculated to be 380. The sample size was calculated based on the sample size formula (i.e., sample size = 4 pq/d^2^) [15]. A list of girls from randomly selected 10 schools was obtained from the respective headmasters and principals of the high schools. Probability proportional to size (PPS) was applied. Adolescent girls from each school were then selected through simple random sampling. Adolescent girls enrolled in rural higher primary schools and rural high schools who had attained menarche, had menarche and had menstrual cycles for at least one year, and were not on any medications or having a chronic illness were included in the study. Adolescent girls who had been diagnosed with primary amenorrhea or with previously diagnosed mental health abnormalities were excluded from the study.

Data collection

Data were collected using the interview technique by PTS, who had previous experience using these questionnaires. Each interview did not last more than 15 minutes per candidate. Interviews were taken in the school. Regarding the sociodemographic profile, a pretested, semi-structured questionnaire was used. To assess depression, Beck's Depression Inventory (BDI-II) was employed. The BDI-II is a Likert scale. The highest possible total for the entire test would be 63, and the lowest possible score for the test would be 0. Each of the 21 items corresponding to a symptom of depression was summed to give a single score for the BDI-II. Each item was rated on a four-point scale ranging from 0 to 3. Two items (16 and 18) had seven options to indicate either an increase or decrease in appetite and sleep. The cut-off score guidelines for the BDI-II are given with the recommendation that thresholds be adjusted based on the characteristics of the sample and the purpose of the BDI-II. A total score of 0-13 is considered a minimal range, 14-19 is mild depression, 20-28 is moderate depression, and 29-63 is severe depression [16]. The Hamilton Anxiety Scale (HAM-A) was used for assessing anxiety. The scale consists of 14 items, each defined by a series of symptoms, and measures both psychic anxiety (mental agitation and psychological distress) and somatic anxiety (physical complaints related to anxiety). Each item is scored on a scale of 0 (not present) to 4 (severe), with a total score range of 0-56, where more than 17 indicates mild severity, 18-24 signifies moderate severity and 25-30 denotes moderate severity [17]. The Premenstrual Symptoms Screening Tool for Adolescents (PSST-A) was used, which reflects and ‘translates’ categorical DSM-IV criteria into a rating scale with degrees of severity. PSST-A measures the severity and impact of premenstrual symptoms, and it is less time-consuming and more practical than other screening tools. PSST-A uses American College of Obstetricians and Gynecologists (ACOG) guidelines for diagnosing PMT and PMDD. The PSST-A questionnaire picks the symptoms that are not relieved within four days of the onset of menstruation, without recurrence until at least the 13th day of the cycle, and present in the absence of any pharmacologic therapy, hormone ingestion, or drug use. The symptoms must reproducibly occur during two cycles of prospective recording. The patient must exhibit an identifiable dysfunction in social, academic, or work performance. The copyright for the PSST-A was purchased from Milo Publishers Canada [18].

Data analysis

All the data entered in an Excel sheet (Microsoft Corporation, Redmond, Washington, United States) were analysed using IBM SPSS Statistics for Windows, Version 22.0 (Released 2013; IBM Corp., Armonk, New York, United States). Descriptive statistics were applied wherever needed, and to compare between groups, a t-test, and ANOVA were used. To check for the association between various factors with PMT, PMDD, and anxiety and depression, chi-square was applied, with the level of significance defined as a p-value less than 0.05. Pearson’s correlation applied to correlate between anxiety and depression with PMT and PMDD. Regression analysis was performed to determine the predictors.

Operational definitions

A nuclear family is defined as a couple and their dependent children, regarded as a basic social unit and a joint family is defined as an extended family, typically consisting of three or more generations and their spouses, living together as a single household.

## Results

Out of 430 rural adolescent girls, 180 (41.9%) were 15 years old, 205 (47.7%) were studying in the ninth standard, 277 (64.4%) belonged to a nuclear family, 392 (91.2%) had a mixed diet, 236 (54.9%) exercised less than 30 minutes per day, 277 (64.4%) had a sleep duration of more than eight hours per day, 144 (33.5%) had menarche at the age of 13 years, 288 (67%) had regular cycles, 266 (61.9%) had a moderate flow during the menstrual cycle, 302 (70.2%) had a cycle duration of less than seven days, and 243 (56.5%) had dysmenorrhea. Out of 430 participants, 38 (8.8%) had PMDD and 75 (17.4%) had PMS. Seventy-seven (18%) had severe depression and 66 (15.3%) had moderate depression according to the BDI-II. With regard to anxiety, 32 (7.4%) had mild to moderate anxiety and 14 (3.3%) had moderate to severe anxiety according to the HAM-A (Table 1).

**Table 1:** Distribution of participants according to clinico-sociodemographic details

Clinico-sociodemographic details		Frequency	Percentage
Age (in years)	12	15	3.5
	13	46	10.7
	14	121	28.1
	15	180	41.9
	16	68	15.8
Class studying in	8	56	13.0
	9	205	47.7
	10	169	39.3
Type of family	Nuclear	277	64.4
	Joint	153	35.6
Diet type	Vegetarian	38	8.8
	Mixed	392	91.2
Exercise duration per day	Less than 30 minutes	236	54.9
	More than 30 minutes	194	45.1
Sleep duration per day	Less than 8 hrs	153	35.6
	More than 8 hrs	277	64.4
Age of menarche	9	33	7.7
	10	2	0.5
	11	40	9.3
	12	107	24.9
	13	144	33.5
	14	90	20.9
	15	14	3.3
Cycle regularity	Regular	288	67.0
	Irregular	142	33.0
Menstrual cycle flow	Mild	113	26.3
	Moderate	266	61.9
	Severe	51	11.9
Duration of the cycle	Less than 5 days	302	70.2
	More than 5 days	128	29.8
Dysmenorrhea	Present	243	56.5
	Absent	187	43.5
Depression	Minimal	235	54.6
	Mild	52	12.1
	Moderate	66	15.3
	Severe	77	18.0
Anxiety	Mild	384	89.3
	Mild to moderate	32	7.4
	Moderate to severe	14	3.3
Premenstrual syndrome	Present	75	17.4
	Absent	355	82.6
Premenstrual dysphoric disorder	Present	38	8.8
	Absent	392	91.2

Of the participants with PMDD, 28 (11.3%) were older than 14 years, 19 (12.4%) belonged to joint families, 10 (19.6%) had severe flow, 24 (18.8%) had a cycle duration of more than seven days, and 28 (11.5%) had dysmenorrhea, 15 (19.5%) had severe depression, and 13 (28.3%) had anxiety. This association between PMDD and factors such as age (in years), type of family, menstrual cycle flow, duration of the cycle, dysmenorrhea, depression, and anxiety was statistically significant, with a p-value of less than 0.05 (Table 2).

**Table 2:** Association between premenstrual dysphoric disorder and clinico-sociodemographic factors *Chi-square Test

Clinico-sociodemographic factors		Premenstrual dysphoric disorder		p-value*
		Present, n (%)	Absent, n (%)	
Age (in years)	Less than 14	10 (5.5%)	172 (94.5%)	0.03
	More than 14	28 (11.3%)	220 (88.7%)	
Class studying in	8th	1 (1.8%)	55 (98.2%)	0.11
	9th	22 (10.7%)	183 (89.3%)	
	10th	15 (8.9%)	154 (91.1%)	
Type of family	Nuclear	19 (6.9%)	258 (93.1%)	0.04
	Joint	19 (12.4%)	134 (87.6%)	
Diet type	Vegetarian	2 (5.3%)	36 (94.7%)	0.4
	Mixed	36 (9.2%)	356 (90.8%)	
Cycle regularity	Regular	21 (7.3%)	267 (92.7%)	0.1
	Irregular	17 (12.0%)	125 (88.0%)	
Menstrual cycle flow	Low	7 (6.2%)	106 (93.8%)	0.01
	Moderate	21 (7.9%)	245 (92.1%)	
	Severe	10 (19.6%)	41 (80.4%)	
Duration of the cycle	Less than 7 days	14 (4.6%)	288 (95.4%)	0.001
	More than 7 days	24 (18.8%)	104 (81.3%)	
Dysmenorrhea	Present	28 (11.5%)	215 (88.5%)	0.001
	Absent	10 (5.3%)	177 (94.7%)	
Exercise duration per day	Less than 30 minutes	22 (9.3%)	214 (90.7%)	0.2
	More than 30 minutes	16 (8.2%)	178 (91.8%)	
Sleep duration per day	Less than 8 hours	16 (10.5%)	137 (89.5%)	0.3
	More than 8 hours	22 (7.9%)	255 (92.1%)	
Age of menarche	Less than 12 years	17 (9.3%)	165 (90.7%)	0.7
	More than 12 years	21 (8.5%)	227 (91.5%)	
Depression	Minimal	12 (5.1%)	223 (94.9%)	0.002
	Mild	4 (7.7%)	48 (92.3%)	
	Moderate	7 (10.6%)	59 (89.4%)	
	Severe	15 (19.5%)	62 (80.5%)	
Anxiety	Absent	25 (6.5%)	359 (93.5%)	0.001
	Present	13 (28.3%)	33 (71.7%)	

Of the participants with PMS, 23% were more than 14 years old, 22.5% were studying in the 10th standard, 23.9% had irregular cycles, 31.4% had severe flow during menstruation, 27.3% had a cycle duration of more than seven days, 23% were suffering from dysmenorrhea, 27.3% had depression, and 37% had anxiety. This association between PMS and factors such as age (in years), class in which studying, cycle regularity, flow during the menstrual cycle, duration of the cycle, dysmenorrhea, depression, and anxiety was statistically significant, with a p-value of less than 0.05 (Table 3).

**Table 3:** Association of premenstrual syndrome with clinico-sociodemographic factors * Chi-square Test

Clinico-sociodemographic factors		Premenstrual syndrome		p-value*
		Present, n (%)	Absent, n (%)	
Age (in years)	Less than 14	18 (9.9%)	164 (90.1%)	0.001
	More than 14	57 (23.0%)	191 (77.0%)	
Class studying in	8th	4 (7.1%)	52 (92.9%)	0.02
	9th	33 (16.1%)	172 (83.9%)	
	10th	38 (22.5%)	131 (77.5%)	
Type of family	Nuclear	44 (15.9%)	233 (84.1%)	0.2
	Joint	31 (20.3%)	122 (79.7%)	
Diet type	Vegetarian	5 (13.2%)	33 (86.8%)	0.4
	Mixed	70 (17.9%)	322 (82.1%)	
Cycle regularity	Regular	41 (14.2%)	247 (85.8%)	0.01
	Irregular	34 (23.9%)	108 (76.1%)	
Menstrual cycle flow	Low	14 (12.4%)	99 (87.6%)	0.01
	Moderate	45 (16.9%)	221 (83.1%)	
	Severe	16 (31.4%)	35 (68.6%)	
Duration of the cycle	Less than 7 days	40 (13.2%)	262 (86.8%)	0.001
	More than 7 days	35 (27.3%)	93 (72.7%)	
Dysmenorrhea	Present	56 (23.0%)	187 (77.0%)	0.001
	Absent	19 (10.2%)	168 (89.8%)	
Exercise duration per day	Less than 30 minutes	39 (16.5%)	197 (83.5%)	0.5
	More than 30 minutes	36 (18.6%)	158 (81.4%)	
Sleep duration per day	Less than 8 hrs	33 (21.6%)	120 (78.4%)	0.09
	More than 8 hrs	42 (15.2%)	235 (84.8%)	
Age of menarche	Less than 12 years	29 (15.9%)	153 (84.1%)	0.4
	More than 12 years	46 (18.5%)	202 (81.5%)	
Depression	Minimal depression	33 (14.0%)	202 (86.0%)	0.001
	Mild depression	10 (19.2%)	42 (80.8%)	
	Moderate depression	11 (16.7%)	55 (83.3%)	
	Severe depression	21 (27.3%)	56 (72.7%)	
Anxiety	Absent	58 (15.1%)	326 (84.9%)	0.001
	Present	17 (37.0%)	29 (63.0%)	

Comparing the duration of the cycle with PMS, those adolescent girls with a duration of more than seven days had higher odds, i.e. 1.96 (95%CI 1.03-3.61) of having PMS and this association was statistically significant. However, while other factors like nuclear family, vegetarian diet, regular cycles, and exercising less than 30 minutes also had higher odds of having PMS, this association was not statistically significant (Table 4).

**Table 4:** Binary logistic regression analysis between anxiety, depression, and premenstrual syndrome BDI: Beck's Depression Inventory

	Beta Coefficient	Standard error	p-value	Odds ratio	95% CI for EXP (B)	
					Lower	Upper
Standard			0.422			
8th	0.863	0.660	0.191	2.370	0.650	8.639
9th	0.161	0.309	0.603	1.174	0.641	2.152
Nuclear family	0.289	0.288	0.315	1.335	0.760	2.345
Vegetarian	0.095	0.524	0.855	1.100	0.394	3.072
Regular	0.496	0.294	0.091	1.643	0.923	2.922
Flow			0.981			
Mild	0.032	0.520	.951	.969	.349	2.684
Moderate	0.037	0.432	.932	1.037	.445	2.418
Cycle duration of more than 7 days	0.661	0.319	.038	1.936	1.037	3.615
Dysmenorrhea present	0˗.849	0.327	0.009	0.428	0.226	0.812
Exercise duration less than 30 minutes	0.258	0.299	0.389	1.294	.720	2.325
Sleep duration less than 30 minutes	0.446	0.311	0.152	.640	.348	1.178
Anxiety present	0.576	0.422	0.172	1.779	.778	4.071
BDI			0.199			
Mild depression	0.516	0.416	0.215	1.675	0.742	3.782
Moderate depression	0.419	0.518	0.419	0.658	0.238	1.816
Severe depression	0.339	0.475	0.475	1.404	0.553	3.561
Constant	0.192	0.676	0.777	0.825		

Comparing the duration of the menstrual cycle with PMDD, it was seen that participants with a duration of more than seven days had higher odds, i.e. 3.02 (95%CI 1.3-6.8) of having PMDD and this association was statistically significant. Comparing anxiety status with PMDD, participants with anxiety had higher odds, i.e. 2.9 (95%CI 1.1-7.6) of having PMDD and this association was statistically significant. While factors like class in which studying, type of family, regular menstrual cycles, and various depression statuses like mild, moderate, and severe also had high odds of having PMDD, this association was not statistically significant (Table 5).

**Table 5:** Binary logistic regression analysis between anxiety, depression, and premenstrual dysmorphic disorder BDI: Beck's Depression Inventory

	B	p-value	Odds ratio	95%CI for EXP (B)	
				Lower	Upper
Standard		0.276			
9th	1.639	0.127	5.149	0.627	42.272
10th	0.066	0.865	0.936	0.436	2.009
Nuclear family	0.631	0.091	1.879	0.905	3.901
Vegetarian	0.418	0.590	1.519	0.332	6.959
Regular	0.229	0.555	1.258	0.587	2.695
Flow		0.894			
Normal	0.257	0.685	1.293	0.374	4.470
Moderate	0.226	.658	1.253	0.461	3.411
Cycle duration of more than 7 days	1.107	.008	3.025	1.335	6.854
Dysmenorrhea	0˗.283	.507	.753	0.327	1.738
Anxiety present	1.068	.030	2.909	1.106	7.653
BDI		0.563			
Mild depression	0.686	0.196	1.986	0.702	5.619
Moderate depression	0.246	0.718	1.279	0.336	4.870
Severe depression	0.547	0.321	1.728	0.586	5.099
Menarche duration of less than 7 days	0.312	0.455	1.366	0.603	3.095
Constant	0.356	0.635	0.700		

## Discussion

Four hundred and thirty rural adolescent girls took part in this study from 20 schools. The majority of them were aged 15 years (n=180, 41.9%), studying in the ninth standard (n=205, 47.7%), belonged to nuclear families (n=277, 64.4%), exercised less than 30 minutes a day (n=236, 54.9%), had a sleep duration of more than eight hours per day (n=277, 64.4%), and had menarche at the age of 13 years (n=144, 33.5%). Among the 430 participants, 67% had regular cycles, 70.2% had a cycle duration of less than seven days, and 56.5% had dysmenorrhea.

Menstrual health concerns can create an extreme physical and psychosocial impact on adolescent girls. Adequate menstrual health is crucial for their healthy physical and mental development [19]. Various studies support the observations of the present cross-sectional study. For instance, a study conducted by Ko et al. showed that women with PMDD had a premenstrual exacerbation of symptoms of mental health [20]. Depression was the most prominent feature of the PMDD diagnosis, whereas irritability was most frequently associated with functional impairment. A study undertaken by Yen et al. found that women with PMDD were more likely to have generalized anxiety disorder (GAD) and that women with GAD had higher odds of having PMDD [21]. Anxiety, depression, and irritability symptoms in women with PMDD and GAD were found to be extremely common and the study suggests that depression, irritability, and behaviour inhibition mediated the link between PMDD, and GAD was also present. In a study conducted by Yang et al., 20% of adolescents reported suffering from distressing premenstrual symptoms, and girls with PMDD and subthreshold PMDD were highly similar in their symptom severity and characteristics [22].

A study by Itriyeva showed that PMS was more common than PMDD and affected 20-40% of menstruating women with typical symptoms such as fatigue, irritability, mood swings, depression, abdominal bloating, breast tenderness, acne, changes in appetite, and food cravings [23]. PMDD, which affects a smaller percentage of women, is characterized by more severe symptoms, and it is listed as a depressive disorder in the Diagnostic and Statistical Manual of Mental Disorders, Fifth Edition (DSM-5) [23,24]. These findings were similar to that of the current study. The results are strongly in line with the fact that the symptoms of PMS and its consequences are much more prevalent.

A study conducted by Mohamadirizi and Kordi showed that a high percentage of students suffer from psychological disorders; it also indicated a significant link between menstruation signs and anxiety, depression, and stress [25]. A longitudinal study undertaken in Uganda among adolescents regarding menstrual characteristics and menstrual anxiety similarly revealed that menstruation caused substantial anxiety [26]. It also showed that menstrual problems had a clinically and statistically significant association with depression among adolescent girls aged 9-11 years. The fact that the increased prevalence of depression correlates with hormonal changes in women, particularly during puberty, prior to menstruation, suggests that female hormonal fluctuations may be a trigger for depression [27]. A study undertaken in China among adolescent girls suggested that menstrual problems and daytime sleepiness were prevalent in adolescent girls possibly because of menstrual irregularities and menstrual pain [28]. The study implied that menstrual health and sleep have a significant impact on the overall health of adolescents. All these studies establish the role of menstrual health in adolescence as a significant factor influencing mental health status, which can be associated with the symptoms of anxiety and depression. Poor menstrual health has been associated with serious ill health, including reproductive tract and urinary tract infections, and its impact on the quality of life cannot be neglected.

Strengths, limitations, and recommendations

The strength of the present study is that validated tools are used for diagnosing all the conditions, namely PMS, PMDD, anxiety, and depression. This study allows for correlating PMS with anxiety and depression scores and predicting the relationship between the physiological phenomenon of menstruation and its relationship with mental health issues among adolescent girls. The study was conducted among adolescent girls in rural areas where access to healthcare was either very minimal or non-existent. One of the limitations of the study is the temporal association and causal effect relationship between anxiety, and depression with PMS and PMDD could not be established. This study recommends that schools be instrumental in designing and implementing menstrual health sessions for adolescent girls on a regular basis. This approach can break the chain of continuity of menstrual health problems and their adverse effects on mental health during this transition to the adult phase. Female school teachers can be trained with pre-structured modules to tackle these neglected problems of adolescent health.

## Conclusions

This study helps to understand the complex interlinked scenarios like mental health issues and their association with menstrual problems, consequently confirming the need to advocate for the timely identification of conditions for promotive and preventive care, which can be started at schools. Girls with problematic periods may be prone to more frequent and severe mental health disorders, which may be associated with a poor quality of life in adolescence and also with significant negative consequences later in life. PMS and PMDD can also be potential risk factors for other mental health issues in this age group, and their early diagnosis can greatly help in the treatment of mental illness. The study shows that varied physiological manifestations of menstrual cycles should be kept in mind by clinicians and a thorough history must be taken regarding the menstrual cycle of adolescent girls during mental health evaluation and treatment. School teachers can be trained in the management of this common health issue and be positive catalysts in influencing the health of adolescent girls. 
